# FHL2 Regulates Natural Killer Cell Development and Activation during *Streptococcus pneumoniae* Infection

**DOI:** 10.3389/fimmu.2017.00123

**Published:** 2017-02-13

**Authors:** Thomas Baranek, Eric Morello, Alexandre Valayer, Rose-France Aimar, Déborah Bréa, Clemence Henry, Anne-Gaelle Besnard, Emilie Dalloneau, Antoine Guillon, Pierre-François Dequin, Emilie Narni-Mancinelli, Eric Vivier, Fabrice Laurent, Yu Wei, Christophe Paget, Mustapha Si-Tahar

**Affiliations:** ^1^INSERM, Centre d’Etude des Pathologies Respiratoires (CEPR), UMR 1100, Tours, France; ^2^Université François Rabelais, Tours, France; ^3^Service de Réanimation Polyvalente, Centre Hospitalier Régional Universitaire, Tours, France; ^4^Centre d’Immunologie de Marseille-Luminy, Aix Marseille Université, INSERM, CNRS, Marseille, France; ^5^Hôpital de la Timone, Assistance Publique-Hôpitaux de Marseille, Marseille, France; ^6^ISP, INRA, Université Tours, Nouzilly, France; ^7^Hépacivirus et immunité innée, Institut Pasteur, Paris, France

**Keywords:** NK cell, maturation, FHL2, transcriptional factor, pneumococcal infections, mouse models

## Abstract

Recent *in silico* studies suggested that the transcription cofactor LIM-only protein FHL2 is a major transcriptional regulator of mouse natural killer (NK) cells. However, the expression and role of FHL2 in NK cell biology are unknown. Here, we confirm that FHL2 is expressed in both mouse and human NK cells. Using FHL2^−/−^ mice, we found that FHL2 controls NK cell development in the bone marrow and maturation in peripheral organs. To evaluate the importance of FHL2 in NK cell activation, FHL2^−/−^ mice were infected with *Streptococcus pneumoniae*. FHL2^−/−^ mice are highly susceptible to this infection. The activation of lung NK cells is altered in FHL2^−/−^ mice, leading to decreased IFNγ production and a loss of control of bacterial burden. Collectively, our data reveal that FHL2 is a new transcription cofactor implicated in NK cell development and activation during pulmonary bacterial infection.

## Introduction

Natural killer (NK) cells are innate lymphocytes involved in tumor recognition, hematopoietic allograft rejection, pregnancy, and control of microbial infections (1). NK cells protect the host directly through the production of cytotoxic effectors, such as perforin and granzymes. To sense target cells, NK cells are educated during development and possess a large panel of antigen-specific receptors. NK cell development takes place in the bone marrow (BM) after birth and is supported by stromal cells through receptor–ligand interactions and the production of cytokines and growth factors (2, 3). Many transcription factors guide the process of NK cell genesis, which is characterized by the sequential acquisition of an array of cell-surface molecules that define distinct NK cell subsets (4–6). Mature NK cells are mostly found in the spleen, lymph nodes, lung, liver and blood, where they exert their cytotoxic immune functions. NK cells also participate in shaping immune responses through the production of cytokines, such as IFNγ and TNFα and through their crosstalk with other immune cells (7). Specifically, by producing IFNγ, NK cells play a critical role in the control of several bacterial infections, including pneumonia (8–10). Thus, during *Streptococcus pneumoniae*-triggered lung infection, NK cells are one of the major cells responsible for IFNγ production. IFNγ production correlates with the lung infiltration and activation of neutrophils and is required for protection against *S. pneumoniae* (11–13).

Four-and-a-half LIM-only protein 2 (FHL2) belongs to the LIM-only protein family. LIM domains are double zinc finger motifs that mediate protein–protein interactions. FHL2 is highly conserved among species and plays important roles in cell proliferation, apoptosis, and signal transduction (14, 15). In the cytoplasm, FHL2 can also interact with integrins and signaling intermediates, such as MAPKs and TRAF-6 (16, 17). Moreover, upon cell activation, FHL2 can rapidly translocate to the nucleus, where it exerts transcriptional cofactor activities that regulate the activity of major transcription factors, such as NF-κB, AP-1, and Foxo1 (18–20). Moreover, FHL2 has been implicated in several immune and inflammatory diseases, such as arthritis and vascular restenosis (21, 22). FHL2 is also involved in lung inflammation, including asthma, fibrosis, and influenza A virus propagation (23–25).

Interestingly, a study using *in silico* analysis cited FHL2 as a protein that could modulate more than 50% of the known NK cell fingerprint (26). Using microarrays data and a network modeling approach, the authors identified 93 genes preferentially expressed in resting NK cells and putative transcriptional regulators of these genes. FHL2 was predicted to be a major regulator of those genes as well as well-known transcriptional factors, such as Tbx21, Eomes, or Stat5. Our present study provides new evidence that FHL2 is expressed in human and mouse NK cells and participates in NK cell development. Using *S. pneumoniae* pulmonary infection and FHL2^−/−^ mice (27), we showed that the activation of lung NK cells is altered in FHL2^−/−^ mice. We also found that FHL2 is a major mediator of IFNγ production during *S. pneumoniae* infection, leading to an impaired neutrophil-mediated immune response, a loss of control of the bacterial burden, and, finally, to an enhanced animal mortality when FHL2 is absent. Thus, the transcription cofactor FHL2 is implicated in NK cell development and in the capacity of NK cells to regulate the antibacterial immune response.

## Results

### FHL2 Expression in Human and Mouse NK Cells

**Figure 1:**
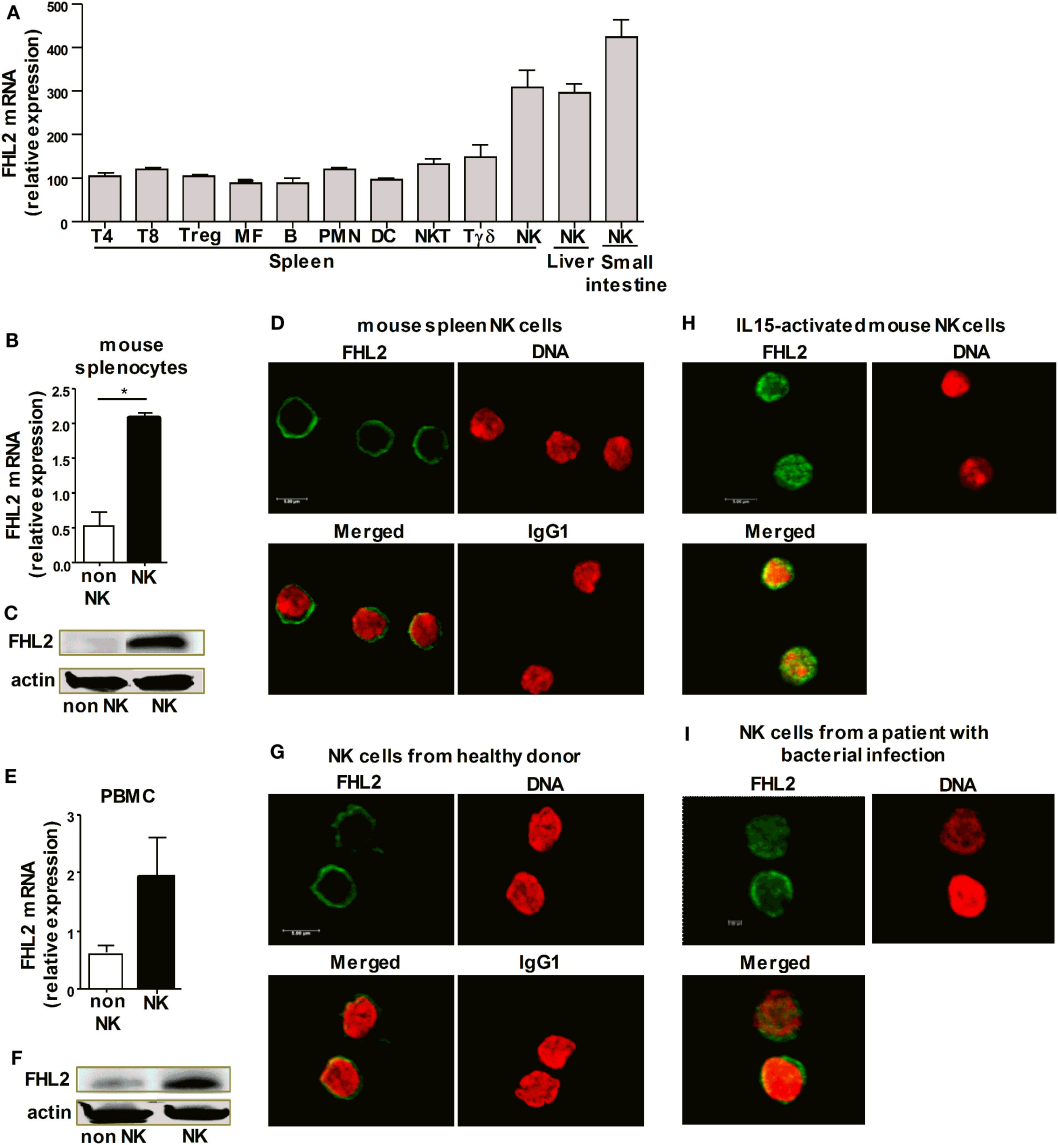
**FHL2 expression in human and mouse natural killer (NK) cells**. **(A)** Genome-wide expression analysis was performed on mouse cells using raw microarray data generated by the Immgen Consortium. The list of all Gene Expression Omnibus accession numbers and corresponding cell populations and series is available in Table S1 in Supplementary Material. **(B–D,H)** NK cells were purified from wild-type mouse spleens. **(E–G)** NK cells were purified from the peripheral blood of healthy donors. **(B,E)** FHL2 mRNA was analyzed using RT quantitative PCR and normalized to GAPDH mRNA in purified NK cells and in non-NK cells. The data are shown as the means ± SEM of at least three independent experiments. *p < 0.05 using the Mann–Whitney test. **(C,F)** Western blot analysis of NK cell lysates. Data are representative of three experiments. **(H)** NK cells were stimulated for 30 min with rmIL-15. **(I)** NK cells were purified from the peripheral blood of patients with a severe bacterial Community-Acquired Pneumonia. **(D,G–I)** FHL2 protein expression was assessed by immunofluorescence using an anti-FHL2 antibody, and DRAQ5™ was used to detect dsDNA. These panels show representative staining of at least two independent experiments.

The transcription cofactor FHL2 was predicted *in silico* to regulate resting NK cells (26). We first addressed the question of whether NK cells express FHL2 at the mRNA and protein level. Based on global mining of the Gene Expression Omnibus (GEO) database, we analyzed the enrichment of FHL2 in different mouse NK cell populations in comparison to other leukocyte subsets. Mouse NK cells from the spleen, liver, and small intestine were found to express FHL2 mRNA (Figure 1A). We confirmed these results by showing that FHL2 mRNA is expressed in NK cells sorted from mouse spleen (Figure 1B). We also showed that splenic NK cells express FHL2 protein in their cytoplasm at steady-state (Figures 1C,D). We, next, examined FHL2 expression in human NK cells. NK cells purified from the peripheral blood of healthy donors expressed FHL2 at both the mRNA level (Figure 1E) and the protein level (Figures 1F,G). As FHL2 is a transcription cofactor known to be localized in the cytoplasm at steady-state and to translocate into the nucleus after activation, we stimulated murine NK cells with rmIL-15 to evaluate the localization of FHL2. In these conditions, immunofluorescence studies showed that FHL2 is translocated into the nucleus of NK cells, whereas it was present in the cytoplasm of resting NK cells (Figure 1H). Interestingly, in NK cells purified from the peripheral blood of patients with bacterial infection, FHL2 was mainly located in the nucleus (Figure 1I). Altogether, these data emphasize that FHL2 is expressed in both mouse and human NK cells.

### NK Cell Development in FHL2^−/−^ Mice

**Figure 2:**
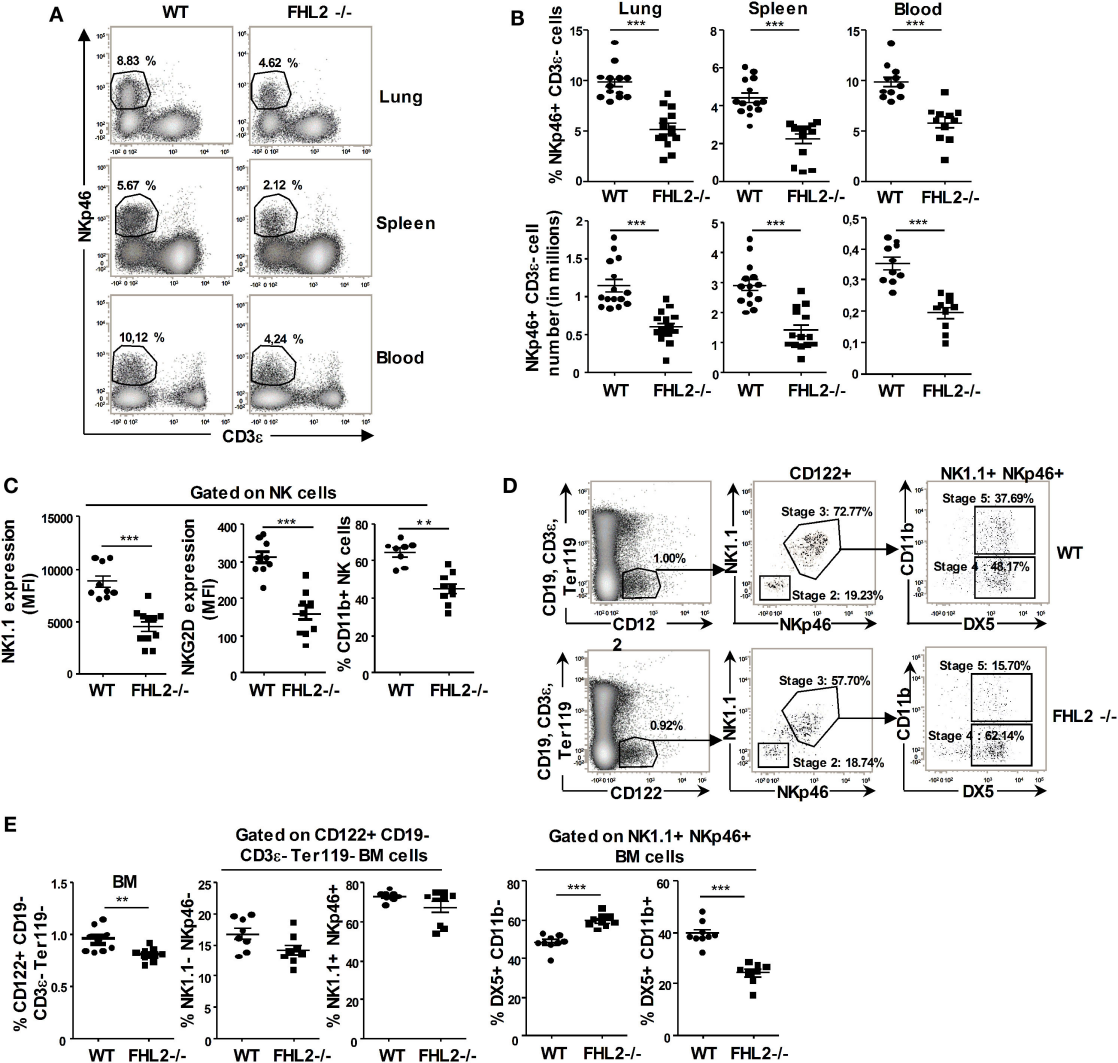
**Natural killer (NK) cell development in FHL2^−/−^ mice**. Flow cytometric analysis of NK cells in various organs of FHL2^−/−^ and wild-type mice. **(A,B)** NK cells were defined as CD19^−^ CD3ϵ^−^ NKp46^+^ cells. **(A)** One representative experiment of the gating in different organs is shown. The percentage of NK cells in each organ is indicated. **(B)** Dots corresponding to the NK cell number and percentage for the indicated organs ± SEM of three experiments (*n* = at least 10 mice) are shown. **(C)** Flow cytometric analysis of NK1.1 and NKG2D expression and the CD11b^+^ percentage within CD19^−^ CD3ϵ^−^ NKp46^+^-gated spleen NK cells ± SEM of two distinct experiments are shown. **(D)** Gating strategy to identify the different stages of NK cell development in the bone marrow (BM). The percentages of cells in each of the specified gates are indicated. **(E)** Dots corresponding to the NK cell frequency for the indicated stage of development in the BM ± SEM of two distinct experiments are shown. **(B,C,E)** Each dot represents the data from one mouse. **(B,C,E)** *p < 0.05, **p < 0.01, ***p < 0.001 by Mann–Whitney test. **(A–C)** Data were confirmed using FHL2^+/+^ littermate mice.

Our data indicate that FHL2 is expressed on NK cells. To decipher the role of this transcription cofactor in NK cells, we next used FHL2-deficient mice (FHL2^−/−^). First, we studied the NK cell compartment in these mice. The relative number and the percentage of NK cells in several peripheral organs, such as the spleen, blood, and lungs, was significantly lower in FHL2^−/−^ mice compared to wild-type (WT) mice (Figures 2A,B). Moreover, the remaining NK cells in FHL2^−/−^ mice displayed an altered phenotype, with lower expression of the surface receptors NK1.1 and NKG2D in the spleen (Figure 2C) and in the lungs (data not shown) than on WT NK cells. Furthermore, monitoring CD11b expression on the NK cell surface allows the study of their maturation status in the peripheral organs (28). In the spleen of FHL2^−/−^ mice, there was a significant reduction of mature CD11b+ NK cells compared with WT mice (Figure 2C). In the BM, precursors committed to the NK-cell lineage express the γ-subunit of the IL-2/IL-15 receptor CD122 and lack other lineage markers. Subsequently, these precursors reach an immature NK-cell phenotype, characterized by the sequential acquisition of NK receptor expression at the cell surface, such as NK1.1 (stage 2), NKp46 (stage 3), DX5 (stage 4), and then CD11b (stage 5) (29). In the BM of FHL2^−/−^ mice, we showed a non-significant decrease in NK cell precursors (CD122^+^ NK1.1^+^) and NK cells at stage 3 (NK1.1^+^ NKp46^+^ NK cells). By contrast, an important difference was observed in the percentage of NK cells at stage 5 (DX5^+^ CD11b^+^ NK cells) between WT mice (39.5% NKp46^+^ NK cells) and FHL2^−/−^ mice (24.3% NKp46^+^ NK cells) (Figures 2D,E). Altogether, these data suggest a role for FHL2 in the development and the maturation of NK cells.

### The NK Cell Defect in FHL2^−/−^ Mice Enhances Their Susceptibility to *S. pneumoniae* Infection

**Figure 3:**
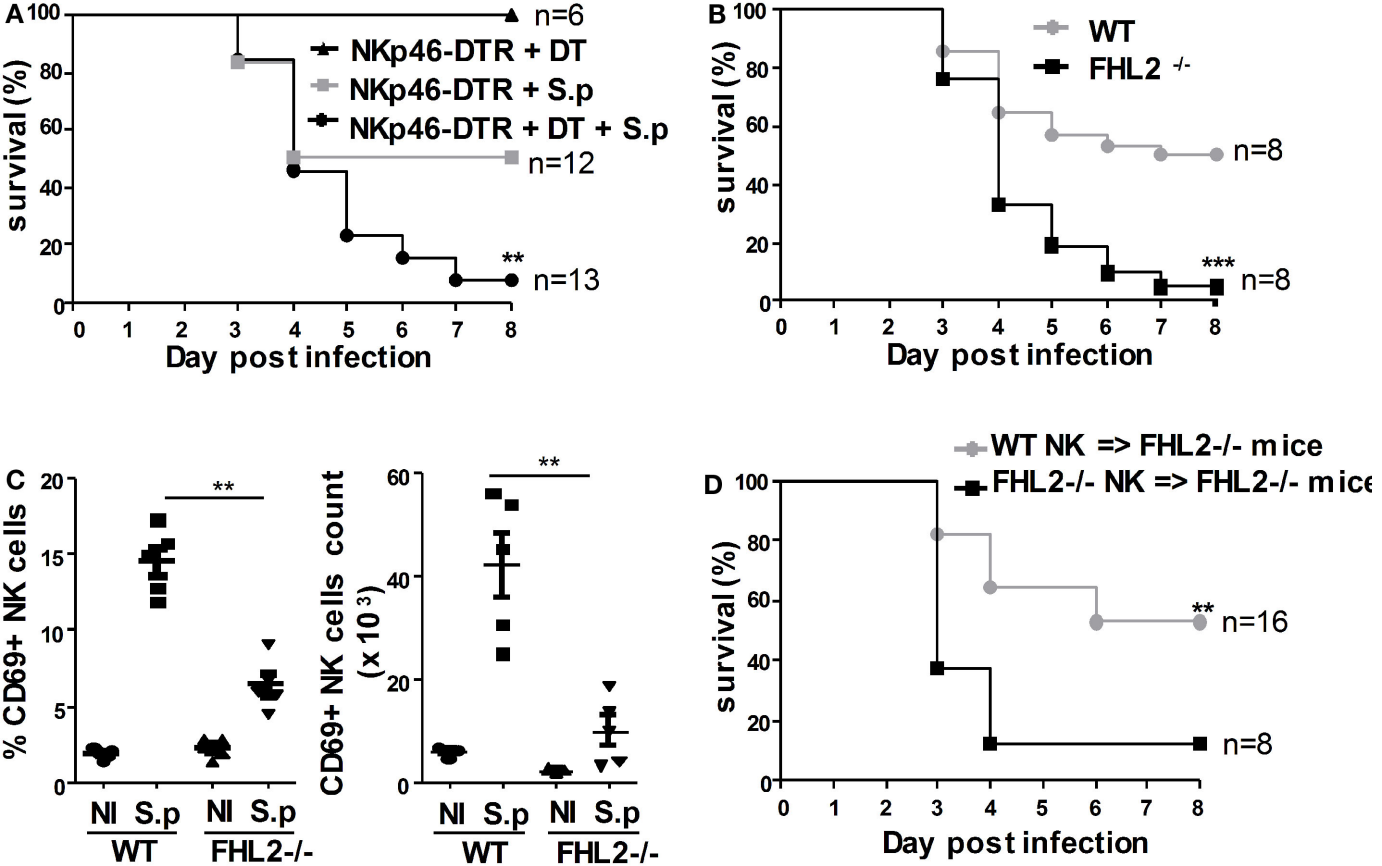
**The natural killer (NK) cell defect of FHL2^−/−^ mice enhances their susceptibility to *Streptococcus pneumoniae* infection**. Mice were infected i.n. with 5 × 10^5^ cfu (LD50) *S. pneumoniae*. **(A)** NKp46(iCre) R26R(DTR) mice were untreated (gray plot) or treated (black circle) with diphtheria toxin (4 ng/g) twice, 24 h before and 24 h after infection. **(B)** FHL2^−/−^ mice (black plot) and wild-type (WT) mice (gray circle) were infected with *S. pneumoniae*. **(C)** Flow cytometric analysis of CD69 expression on lung NK cells. Each dot represents the data obtained for one mouse. Experiment representative of four independent experiments. **p < 0.01 by Mann–Whitney test. **(D)** Purified NK cells from FHL2^−/−^ mice (FHL2^−/−^ NK cells → FHL2^−/−^ mice; black plot) or WT mice (WT NK cells → FHL2^−/−^ mice; gray circle) were i.v. injected in FHL2^−/−^ recipient mice at the time of infection. **(A,B,D)** Survival is shown for two or three independent experiments pooled together. Statistical analysis was performed using the Mantel–Cox test (**p < 0.01, ***p < 0.001). The surviving mice were kept until day 10 postinfection. None died after day 7. **(B)** Data were confirmed using FHL2^+/+^ littermate mice.

Natural killer cells are implicated in innate immune defense during *S. pneumoniae* infection through the production of IFNγ (30, 31). Owing to the large number of pneumococcal serogroups and the possible differences in the associated immune responses, we first confirmed that NK cells contribute to the clearance of *S. pneumoniae* serotype 1 using NKp46(iCre) R26R(DTR) mice. Diphtheria toxin (DT) injection in these mice results in NK cell ablation in the peripheral blood as well as in the spleen, lymph nodes, and lungs (32). Upon infection with LD50 *S. pneumoniae* (5 × 10^5^ cfu) and DT injection, 90% (12 out of 13) of the NKp46(iCre) R26R(DTR) mice died (Figure 3A). In contrast, in the absence of DT injection, 50% (6 out of 12) of the NKp46(iCre) R26R(DTR) mice died (Figure 3A). Hence, mice that were depleted of NK cells had enhanced mortality resulting from *S. pneumoniae* infection.

As we have shown that FHL2^−/−^ mice displayed an NK cell deficiency, we next assessed the behavior of these mice during *S. pneumoniae* infection. Strikingly, 95% (20 out of 21) of the FHL2^−/−^ mice infected with 5 × 10^5^ cfu of *S. pneumoniae* died compared to 50% of the WT mice (Figure 3B). Our previous data indicated a lower NK cell number in FHL2^−/−^ mice compared to WT mice, but there were still NK cells in the lungs of FHL2^−/−^ mice. To further investigate the FHL2-dependent control of *S. pneumoniae*, we next analyzed the activation of the remaining NK cells in the lungs of FHL2^−/−^ mice 24 h following intranasal challenge with the bacteria. CD69 is rapidly expressed at high levels on activated NK cells, including during *S. pneumoniae* infection, and acts as a costimulatory molecule in cytokine secretion (33, 34). Upon *S. pneumoniae* infection, CD69 was less robustly induced on the surface of lung NK cells in FHL2^−/−^ mice compared to WT mice (Figure 3C). Two other activation NK cell markers, CD62L and CD11b, were also less induced on lung NK cells in FHL2^−/−^ mice compared to WT mice (data not shown). It is of note that we did not observe any NK cell proliferation nor apoptosis during the early course of pneumococcal infection in WT mice as well as in FHL2^−/−^ mice (data not shown). Next, we aimed to rescue the susceptibility of FHL2^−/−^ mice to *S. pneumoniae*. We transferred purified FHL2^−/−^ or WT NK cells into FHL2^−/−^ mice at the time of infection. In accordance with the previous results obtained in FHL2^−/−^ mice, upon infection with 5 × 10^5^ cfu *S. pneumoniae*, 88% (7 out of 8) of the FHL2^−/−^ mice that received FHL2^−/−^ NK cells (FHL2^−/−^ NK cells → FHL2^−/−^ mice) died (Figure 3C). In sharp contrast, only 47% (8 out of 17) of the FHL2^−/−^ mice that received WT NK cells (WT NK cells → FHL2^−/−^ mice) died (Figure 3D). Altogether, these data strongly highlight the key functions of FHL2 expression in NK cells during pulmonary infection with *S. pneumoniae*.

### FHL2 Deficiency Decreases *S. pneumoniae*-Induced IFNγ Production by NK Cells

**Figure 4:**
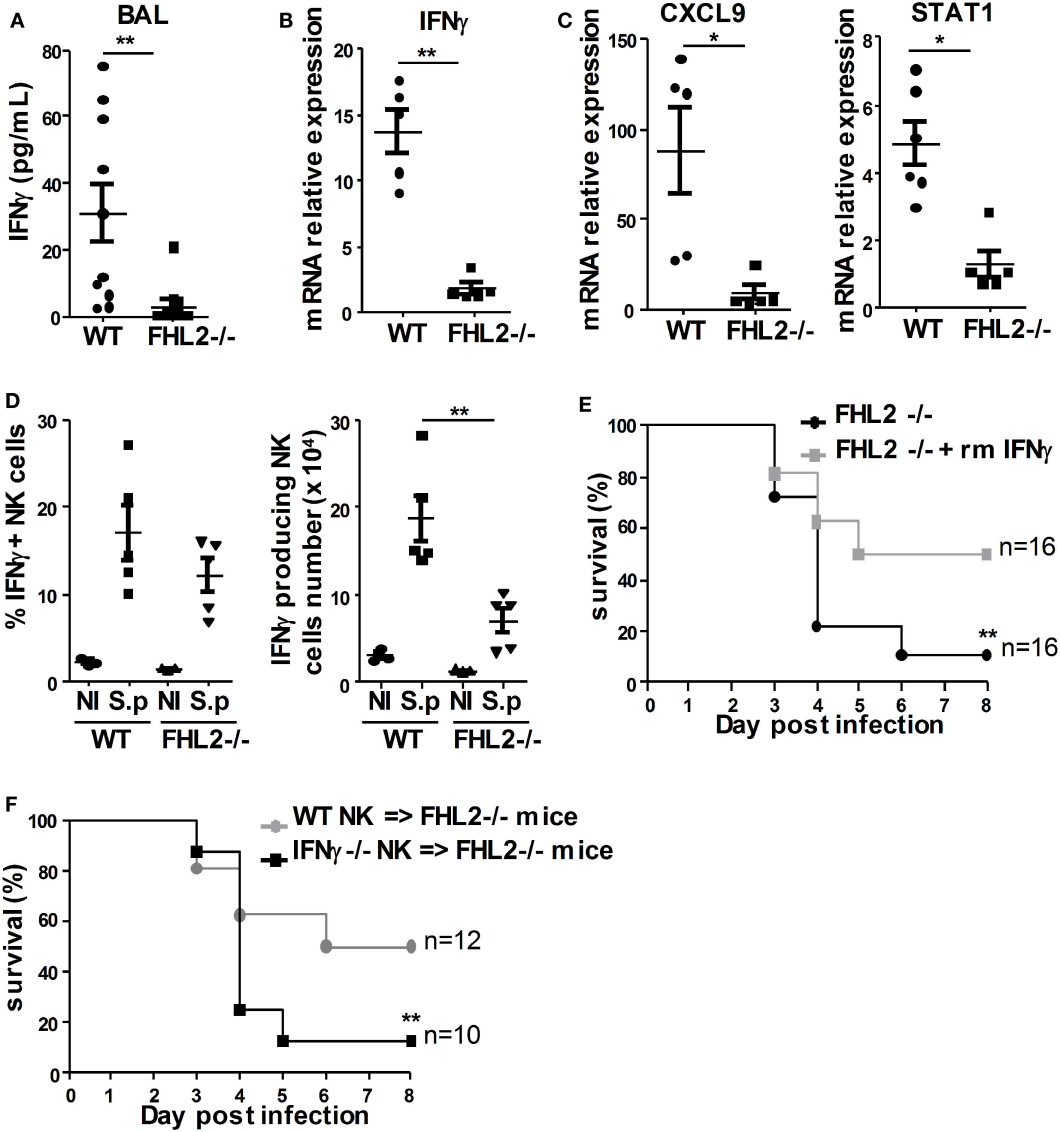
**FHL2 deficiency decreases *S. pneumoniae*-induced IFNγ production by natural killer (NK) cells**. Mice were infected i.n. with 5 × 10^5^ cfu (LD50) *S. pneumoniae*. **(A)** The protein expression of IFNγ was determined by ELISA in the bronchoalveolar lavage obtained from wild-type (WT) and FHL2^−/−^ mice, 24 h postinfection. Results of two distinct experiments are shown. **(B,C)** qRT-PCR was performed to assess the mRNA expression of **(B)** IFNγ and **(C)** CXCL9 and STAT1 in total lungs obtained from WT and FHL2^−/−^ mice, 24 h postinfection. Experiment representative of two independent experiments. **(D)** Intracellular IFNγ levels in lung NK cells were detected using flow cytometry (*n* = 5). **(A–D)** Each dot represents the data from 1 mouse. *p < 0.05, **p < 0.01 by Mann–Whitney test. **(E)** FHL2^−/−^ mice were untreated (black circle) or i.p. injected (gray plot) with rmIFNγ (10 μg/mouse) twice, at the time of infection and 48 h postinfection. **(F)** Purified NK cells from IFNγ^−/−^ mice (IFNγ^−/−^ NK cells → FHL2^−/−^ mice; black plot) or WT mice (WT NK cells → FHL2^−/−^ mice; gray circle) were i.v. injected in FHL2^−/−^ recipient mice at the time of infection. **(E,F)** Survival is shown for two or three independent experiments pooled together. Statistical analysis was performed using the Mantel–Cox test (**p < 0.01). The surviving mice were kept until day 10 postinfection. None died after day 7.

The involvement of IFNγ in the development of pulmonary pneumococcal infection has been studied in detail and has been associated with enhanced clearance of bacteria (12, 35, 36). To further understand the elevated susceptibility of FHL2^−/−^ mice to *S. pneumoniae*, we next analyzed IFNγ production in the bronchoalveolar lavage (BAL) and in the lungs 24 h following intranasal challenge with the bacteria. Local pulmonary IFNγ production was quantified by ELISA for the BAL and by real-time PCR for the lungs of infected mice. In the BAL, IFNγ production was significantly less induced in FHL2^−/−^ mice compared to WT mice upon *S. pneumoniae* infection (Figure 4A). The fold increase of IFNγ transcripts in the lungs after 24 h of infection was also significantly higher in WT mice compared to FHL2^−/−^ mice (Figure 4B). Other IFNγ-related genes, such as CXCL9 and STAT1, are known to be increased during *S. pneumoniae* lung infection (12). We, next, studied the expression of these two genes in the lungs of WT and FHL2^−/−^ mice 24 h after infection. In accordance with the results obtained with IFNγ, the CXCL9 and STAT1 transcripts were less robustly increased in FHL2^−/−^ compared to WT mice (Figure 4C). To further characterize the specific role of NK cells in the defect of IFNγ level in infected FHL2^−/−^ mice, we next analyzed the intracellular production of IFNγ in WT and FHL2^−/−^ lung NK cells following infection. The frequency of IFNγ-producing NK cells in the lung of FHL2^−/−^ mice was reduced compared to their WT counterparts (Figure 4D).

To compensate for the IFNγ defect in FHL2^−/−^ mice infected with *S. pneumoniae*, we next treated those mice with two i.p. injections of recombinant mouse (rm)IFNγ. Interestingly, treatment of FHL2^−/−^ mice with rmIFNγ rescued the phenotype, as only 50% (8 out of 16) of the FHL2^−/−^ mice infected with 5 × 10^5^ cfu *S. pneumoniae* and treated with rmIFNγ died compared to 90% of the untreated FHL2^−/−^ mice (16 out of 18) (Figure 4E). During *S. pneumoniae* infection, different cell types participate in the production of IFNγ, including NK cells, NKT cells, and γδ T cells (11, 37). To test the specific role of NK cells in IFNγ production during *S. pneumoniae* infection, we transferred NK cells purified from IFNγ-deficient mice into FHL2^−/−^ mice before lung infection. When reconstituted with IFNγ-deficient NK cells (IFNγ^−/−^ NK cells → FHL2^−/−^ mice), 88% (7 out of 8) of the FHL2^−/−^ mice died, whereas 50% (5 out of 10) of the FHL2^−/−^ mice reconstituted with WT NK cells (WT NK cells → FHL2^−/−^ mice) survived (Figure 4F). Altogether, we demonstrate that the production of IFNγ by NK cells is impaired in FHL2^−/−^ mice, leading to increased susceptibility to *S. pneumoniae* infection.

### Impaired Antibacterial Immune Response in FHL2-Deficient Mice

**Figure 5:**
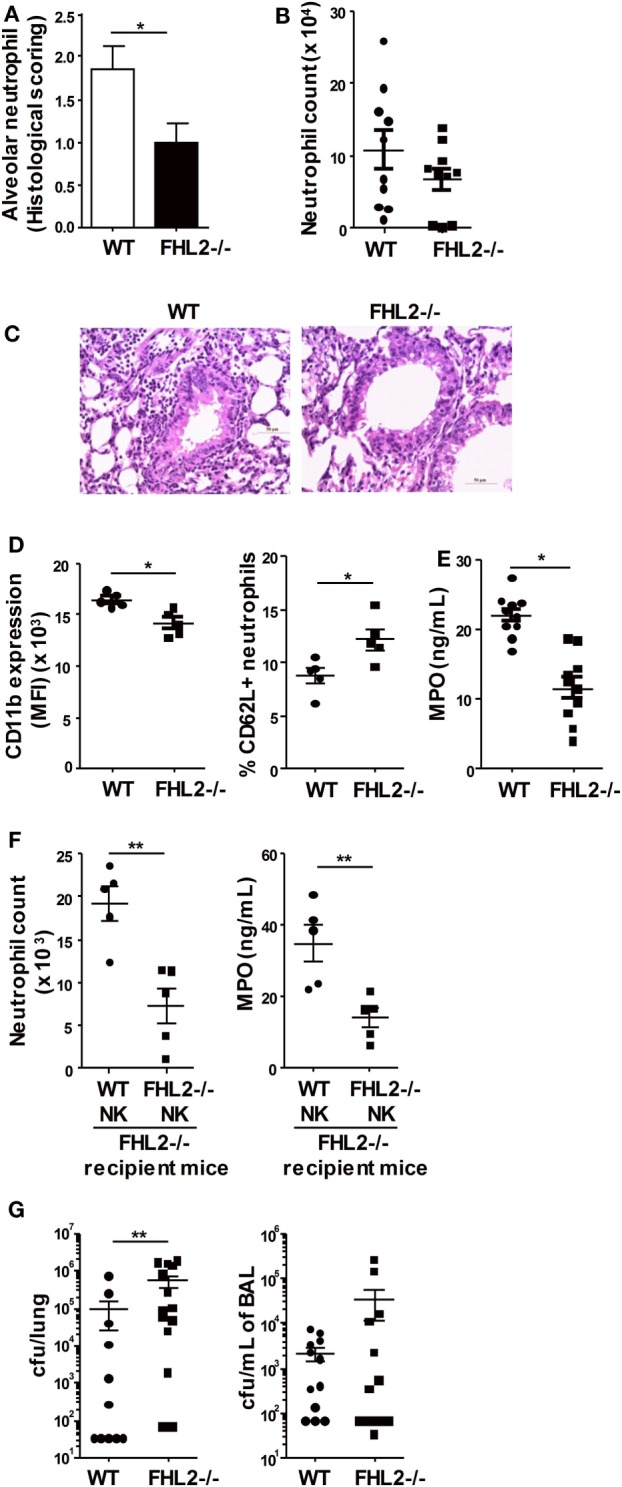
**Impaired antibacterial immune response in FHL2-deficient mice**. Mice were infected i.n. with 5 × 10^5^ cfu (LD50) *S. pneumoniae*. **(A,C)** Histological examination of the lung tissue obtained from wild-type (WT) and FHL2^−/−^ mice 24 h postinfection, stained with hematoxylin-eosin and examined by light microscopy. **(A)** Semi-quantitative pathology scores (described in experimental procedures) for the neutrophil number per lung microscopic field. The data are presented as the means ± SEM of two distinct experiments (*n* = 5 mice per group) are shown. *p < 0.05 by Mann–Whitney test. **(B)** Neutrophil numbers were determined by flow cytometry in the bronchoalveolar lavage (BAL) obtained from WT and FHL2^−/−^ mice 24 h postinfection. **(C)** Representative photomicrographs of lung sections to show neutrophil colonization of WT and FHL2^−/−^ lungs. **(D)** Flow cytometric analysis of CD11b expression and CD62L^+^ percentage on F4/80^−^ Ly6G^high^-gated neutrophils in the BAL obtained from WT and FHL2^−/−^ mice 24 h postinfection. Data are representative of three distinct experiments. **(E)** The protein expression of myeloperoxidase (MPO) was determined by ELISA in the BAL obtained from WT and FHL2^−/−^ mice 48 h postinfection. The mean ± SEM of two experiments is shown. **(F)** Purified natural killer cells from FHL2^−/−^ mice or WT mice were i.v. injected in FHL2^−/−^ recipient mice at the time of infection. Then, neutrophil numbers and MPO expression were determined by flow cytometry and ELISA, respectively, in the BAL 24 h postinfection (*n* = 5). **(G)** Bacterial burden in the lungs and BAL obtained from WT and FHL2^−/−^ mice 48 h postinfection. Data are representative of two distinct experiments. **(B,D–G)** Each dot represents the data from one mouse. *p < 0.05, **p < 0.01 by Mann–Whitney test.

IFNγ production during *S. pneumoniae* infection is involved in the regulation of the neutrophil-mediated host defense against this infection (38, 39). To further characterize the development of the immune response in FHL2^−/−^ mice during *S. pneumoniae* infection, we analyzed the recruitment of neutrophils in the BAL and lung tissue. Histological analysis showed significantly attenuated infiltration of neutrophils in the lungs of FHL2^−/−^ mice compared to WT mice (Figures 5A,C). In accordance with this observation, the neutrophil number in the BAL of FHL2^−/−^ mice was lower than in WT mice 24 h postinfection (Figure 5B). Neutrophil activation is associated with upregulation of CD11b, shedding of CD62L, and degranulation of antibacterial products such as neutrophil myeloperoxidase (MPO) (40). The CD11b fluorescence intensity of neutrophils was lower in the BAL of FHL2^−/−^ mice than in WT mice (Figure 5D). In contrast, the percentage of CD62L^+^ neutrophils was higher in FHL2^−/−^ mice than in WT mice (Fic. 5D). Moreover, the level of MPO in the BAL was significantly lower in FHL2^−/−^ mice than in WT mice (Figure 5E). Interestingly, the neutrophil recruitment and activation defect was compensated by the transfer of purified WT NK cells into FHL2^−/−^ mice at the time of infection (Figure 5F).

As neutrophils are known to play a critical role in the killing of *S. pneumoniae*, we next evaluated the impact of FHL2 deficiency on the clearance of the bacteria. In accordance with the survival results, 48 h after inoculation, the bacterial load in the lungs of FHL2^−/−^ mice was significantly higher than in WT mice (Figure 5G). The number of bacteria in the BAL of FHL2^−/−^ mice was also increased compared to WT mice (Figure 5G). Altogether, these data highlighted the impaired neutrophil-mediated immune response to *S. pneumoniae* in FHL2^−/−^ mice, leading to a defect in bacterial control.

## Discussion

Natural killer cell development, maturation, and functions are controlled by successive and coordinated actions of transcription factors (29, 41). Whole-genome studies contribute to improving the knowledge of the complex NK cell biology and *in silico* analyses pinpoint the potential role of FHL2 as a major transcriptional regulator of the signature genes of resting NK cells (26). Here, we show that FHL2 is expressed in both human and mouse NK cells at the mRNA and protein level. Moreover, FHL2 is expressed in the cytoplasm in resting NK cells and translocates to the nucleus upon activation. These results are consistent with those described in other cell types, such as cardiac muscle cells, in which FHL2 is associated with integrins in the cytoplasm (42) and could also be found in the nucleus acting as a transcriptional cofactor (15). Using FHL2^−/−^ mice, we demonstrated that NK cells *in vivo* are impacted by FHL2 deficiency as those mice display a defect in peripheral NK cell numbers, frequency, and maturation. The absence of FHL2 results in alterations in NK cell progenitors at the early stages of development, making FHL2 a potential cofactor of transcription factors acting during these stages of NK cell development (41). Notably, FHL2 has already been associated with hematopoietic stem cell differentiation. FHL2 is expressed in subsets of hematopoietic progenitor cells, and most recently, FHL2 was identified as a critical modulator of hematopoietic progenitor cell functions under stress conditions (43, 44). Overall, FHL2 is implicated in cell differentiation, and it will be of interest to investigate the potential interaction of FHL2 with transcription factors regulating NK cell development and maturation. Moreover, the issue of intrinsic versus extrinsic effects of FHL2 is unresolved. In that regard, bone marrow chimera experiments would be decisive to determine whether FHL2 induces cell-intrinsic effects in NK cells or acts indirectly through non-hematopoietic cells.

Natural killer cells are key players of the early immune response to *S. pneumoniae* infection, but their contribution to pathogenesis remains unclear and dependent on the *S. pneumoniae* serotype used. Using NK cell depletion models, research has highlighted the essential role of NK cells in the early response to pulmonary *S. pneumoniae* serotype 3 infection (30), although they are involved in pathogenesis in a model of pneumococcal meningitis and in pulmonary infection with a serotype 2 or a clinical specimen of *S. pneumoniae* (11, 31, 45). Using NKp46(iCre) R26R(DTR) mice, in which NK cells may be specifically depleted, we showed in this study that NK cells are indispensable for the early control of the pathogenic strain of *S. pneumoniae* serotype 1, a major serotype associated with invasive disease in humans (46). Moreover, the higher susceptibility of FHL2^−/−^ mice compared to WT mice to *S. pneumoniae* serotype 1 infection is clearly linked to the NK cell defect as the transfer of WT NK cells into FHL2^−/−^ mice rescued their immune protection.

IFNγ production is the main contribution of NK cells during *S. pneumoniae* infection and is critical for protection against this pathogen (12). Our study demonstrates that FHL2 is implicated in IFNγ production during *S. pneumoniae* infection and that treatment with rmIFNγ protected FHL2^−/−^ mice. As a consequence of the IFNγ defect, neutrophil recruitment and activation were lower in FHL2^−/−^ mice compared to WT mice. Moreover, the control of the bacterial burden was impaired in FHL2^−/−^ mice compared to WT mice, leading to the enhanced mortality of these mice. FHL2 has already been implicated in different inflammatory process in mice (21, 22) as well as in humans, where FHL2 was proposed to be a marker of lung fibrosis (47). Recently, loss of FHL2 has been associated with an impaired inflammatory reaction after cardiac ischemia owing to a defect in immune cell migration (48). Consistent with the impaired IFNγ production in FHL2^−/−^ mice, we also found that CXCL9 and STAT1, two IFNγ-related genes that are upregulated during *S. pneumoniae* infection (12), are also less robustly expressed in FHL2^−/−^ infected mice compared to WT mice. Interestingly, CXCL9 is one of the chemokines that recruits CD4^+^ T lymphocytes. Pneumococcal-specific CD4^+^ T lymphocytes have an important role in protection against bacterial carriage and pulmonary infection through the production of IL-17 and IFNγ at late time points (49–51).

Using NK cells purified from IFNγ^−/−^ mice and transferred into FHL2^−/−^ mice, we demonstrated that among all cells that produce IFNγ during *S. pneumoniae* infection, NK cells are the major cell population impacted in those mice and that their defect in IFNγ production is sufficient to weaken the antibacterial immune response. FHL2 can regulate the activity of many signaling pathways, including TRAF6 in osteoclasts, MAPKs in muscle cells and mesenchymal stem cells, and NK-κB in different cell types (18, 52, 53). Interestingly, TRAF6, NF-κB, and the MAPKs are members of the signaling pathways activated in NK cells for IFNγ secretion [for review, see Ref. (54)], and NF-κB activation is crucial during pneumococcal pneumonia (55). Therefore, it will be important in future studies to investigate the interactions of FHL2 with these signaling routes to further decipher the molecular mechanisms leading to IFNγ production in NK cells. The impaired IFNγ production in FHL2^−/−^ mice could also be the consequence of the low number and defective maturation status of NK cells. Indeed, NK cell effector functions are associated with the maturation state of NK cells in peripheral organs, and the activation of NK cells is required for IFNγ production (28). In the lungs of FHL2^−/−^ mice, the proportion of mature CD11b^+^ NK cells at steady-state and CD69^+^ activated NK cells after *S. pneumoniae* infection were lower than that in WT mice.

The transcription cofactor FHL2 regulates numerous cellular processes. In this study, we have identified FHL2 as an important mediator that regulates NK cell development, maturation, and activation. FHL2 deletion has functional consequences as FHL2^−/−^ mice loose their ability to control pulmonary *S. pneumoniae* infection owing to a defect in IFNγ production.

This study paves the way for further investigations to delineate the molecular mechanisms by which FHL2 regulates the physiologic and pathologic states of NK cells.

## Experimental Procedures

### Mice, Treatments, and Cell Line

Wild-type (WT) C57BL/6JRj mice were purchased from Janvier Labs (France). FHL2^−/−^, NKp46(iCre) R26R(DTR), and IFNγ^−/−^ mice are described elsewhere (18, 32, 56). Some experiments using FHL2^−/−^ mice were confirmed using FHL2^−/−^ and FHL2^+/+^ littermates obtained from intercrossing FHL2^+/−^ mice. The animals were used between 7 and 13 weeks of age. All experiments were performed in the animal facilities of Tours University according to guidelines of the ethical committee. For NKp46 cell depletion, NKp46(iCre) R26R(DTR) mice were injected intraperitoneally (i.p.) with DT (4 ng/g, Servibio) twice, 1 day before and 1 day after *S. pneumoniae* infection. For treatment with IFNγ, FHL2^−/−^ mice were injected i.p. with rmIFNγ (10 μg/mouse, Peprotech) twice, on day 0 and day 2 postinfection.

### Bacteria, Infection, and Assessment of Bacterial Counts

*Streptococcus pneumoniae* serotype 1 (clinical isolate E1586) and working stocks were prepared as described previously (57). Mice were anesthetized and administered intranasally (i.n.) with 5 × 10^5^ bacteria (LD50). The mice were monitored every 12 h for illness and mortality for up to 10 days. The bacterial burden in the lungs and BAL samples was measured by plating lung homogenates or BAL samples onto blood agar plates. Colony-forming units were enumerated 24 h later.

### Cell Preparation

Lungs were perfused with 10 mL PBS injected into the heart. Splenocyte and lung suspensions were obtained by mechanical disruption and enzymatic digestion, respectively, using gentleMACS dissociators (Miltenyi Biotech) according to the kit manufacturer’s instructions. Bronchoalveolar lavage (BAL) was performed as described previously (58). Red blood cells were lysed in BD Pharm Lyse™ lysing buffer (BD Biosciences).

### Human Cell Isolation

Blood samples were collected from healthy volunteers from the Etablissement Francais du Sang. Blood samples from patients with a Community-Acquired Pneumonia were obtained from the Intensive Care Unit of the University Hospital of Tours. Human blood NK cells were isolated from PBMC by negative magnetic selection using NK Cell Isolation Kit (Miltenyi Biotec). The study was approved by the French national bioethics authorities (CPP-37 2012-R21).

### Flow Cytometry and Antibodies

Flow cytometric analyses were performed using a MACSQuant^®^ Analyzer (Miltenyi Biotec) and VenturiOne software (AppliedCytometry). The following mAbs were used: FITC-conjugated anti-CD62L (MEL-14), APC-conjugated anti-Ly6G (1A8), FITC-conjugated anti-CD3ϵ (145-2C11), FITC-conjugated anti-CD19 (1D3), PerCP-Cy5.5-conjugated anti-NK1.1 (PK136), PE-conjugated anti-CD122 (TM-beta1), APC-conjugated anti-CD49b (DX5), FITC-conjugated anti-Ter119 (TER-119), and PE-Cy7-conjugated anti-IFNγ (XMG1.2) were from BD Biosciences (East Rutherford, NJ, USA); vioblue-conjugated anti-F4/80 (clone BM8), APC-eFluor780-conjugated anti-CD45 (30-F11), FITC-conjugated anti-CD86 (GL1), PerCP-eFLuor710-conjugated anti-MHC2 (M5/114), eFluor450-conjugated anti-CD335 (NKp46, 29A1.4), PerCP-Cy5.5-conjugated anti-CD11b (M1/70), PE-Cy7-conjugated anti-CD27 (LG.7F9), APC-conjugated anti-CD69 (H1.2F3), PE-Cy7-conjugated anti-CD314 (NKG2D; CX5), and APC-eFluor780-conjugated anti-CD45 (30-F11) were from Affymetrix eBioscience. Dead cells were stained with LIVE/DEAD^®^ Fixable Aqua Dead Cell Stain kit (Molecular Probes).

### Adoptive Transfer of NK Cells

Splenocyte suspensions from WT, FHL2^−/−^, or IFNγ^−/−^ donor mice were obtained by mechanical disruption of the spleens of the mice using gentleMACS dissociators (Miltenyi Biotech). Red blood cells were lysed in BD Pharm Lyse™ lysing buffer (BD Biosciences). The preparations were then enriched in NK cells by negative depletion using a mouse NK cell isolation kit (Miltenyi Biotec). The NK cell purity was at least 70%. Then, 1 × 10^6^ donor NK cells/mouse were injected i.v. into FHL2^−/−^ recipient mice.

### Real-time PCR

Total RNA was extracted from NK cells or from lung tissue using the NucleoSpin RNA extraction kit (Macherey-Nagel). Total RNA was quantified using a Nanodrop 2000c spectrophotometer (Thermo Scientific), and then single-strand cDNA was synthesized from 500 ng total RNA from each sample with the High Capacity cDNA Reverse Transcription kit (Applied Biosystems) according to the manufacturer’s instructions. PCR reactions were prepared with 5 μL of cDNA using SYBR Premix Ex Taq (Takara Bio Inc.) and were performed on a LightCycler 480 (Roche Diagnostics GmbH). The sequences of the primers used in this study are as follows: mouse GAPDH 5′-TCAGATCCACGACGGACACA-3′ and 5′-TGCCCAGAACATCATCCCTG-3′; mouse IFNγ 5′-GTGGGTTGTTGACCTC AAACTAGGC-3′ and 5′-GTCTGAATAACTATTTTAACTCAAG-3′; mouse CXCL9 5′-GGAGTTCGAGGAACCCTAGTG-3′ and 5′-GGGATTTGTAGTGGATCGTGC-3′; mouse STAT1 5′-CGGAGTCGGAGGCCCTAAT-3′and 5′-ACAGCAGGTGCTTCTTAATGAG-3′; mouse FHL2 5′-ATGACTGAACGCTTTGACTGC-3′ and 5′-CGATGGGTGTTCCACACT CC-3′; human FHL2 5′-GTACAGACTGCTATTCCAACGAG-3′ and 5′-GCACT GCATGGCATGTTGTT-3′.

### Immunofluorescence

Approximately 2 × 10^5^ cells were seeded onto Superfrost slides and fixed with 4% formaldehyde in PBS. Non-specific binding sites were blocked by incubation with 10% goat serum and 1% BSA in PBS, and then, the slides were stained with primary mouse anti-FHL2 antibody (F4B2-B11 from Thermo Fisher Scientific) overnight at 4°C. Bound antibodies were detected using FluoProbes488-conjugated anti-IgG secondary antibody (Interchim). DRAQ5™ (Biostatus) was used to detect dsDNA. The samples were analyzed with Leica TCS SP8 confocal microscope and Leica LAS X software available in the microscopy facility of the Tours University.

### Histological Analysis

Lungs were collected in 4% paraformaldehyde in PBS and the lung sections, cut at approximately 4 μm in thickness, were stained with hematoxylin-eosin. A study pathologist examined the tissue sections in a blinded fashion using light microscopy on a Leica Diaplan microscope. All histopathological findings were graded in a semi-quantitative fashion on a scale of 0–4 (0: absent, 1: mild, 2: moderate, 3: severe, 4: very severe). All lung preparations and analyses were performed at the LAPV (Amboise, France).

### ELISA

The concentration of IFNγ and MPO secreted into the BAL were measured using optimized standard sandwich ELISA (R&D systems) according to the kit manufacturer’s instructions.

### Microarray Analysis

Raw Affymetrix.CEL files generated by the Immgen Consortium and corresponding to various immune cell populations were downloaded from the GEO repository (Series GSE75202 and GSE37448).

Quality control and normalization of the expression data by Robust Multi-Array Average (59) was performed through Bioconductor in the R statistical environment (version 3.2.0) using the oligo package (version 1.32.0).

A list of all GEO accession numbers and corresponding cell populations and series is available as Table S1 in Supplementary Material.

### Statistical Analysis

All results are expressed as the means ± SEM. Statistical significance was determined using the Mantel–Cox test or the Mann–Whitney test, depending on the analysis. The data were analyzed using GraphPad Prism 5 (GraphPad Software). We considered *p*-values < 0.05 to be significant (*p < 0.05; **p < 0.01; ***p < 0.001).

## Author Contributions

MS-T and TB conceived the study. CP, MS-T, and TB conceived and designed the experiments, while AV, A-GB, CH, DB, ED, EN-M, R-FA, and TB performed them. EN-M, A-GB, MS-T, and TB were involved in data analysis. EN-M, EV, FL, and YW provided mouse strains. AG and P-FD provided blood from patients with a Community-Acquired Pneumonia. MS-T and TB wrote the paper. All the authors have read and approved the revised manuscript.

## Conflict of Interest Statement

The authors declare that the research was conducted in the absence of any commercial or financial relationships that could be construed as a potential conflict of interest.
