# Supplementary material for: FHL2 Regulates Natural Killer Cell Development and Activation during Streptococcus pneumoniae Infection
Source: Front Immunol. 2017 Feb 13;8:123. doi: 10.3389/fimmu.2017.00123 (PMC5303898; doi:10.3389/fimmu.2017.00123)
Supplement: Supplementary file 1 [file Table_1.PDF]

Supplemental information

Supplementary Table I. Origin of the public microarray data used

GEO accession	cell type	GEO series	Figure 1A legend
GSM1585333	NK.CD49b+.Lv#1	GSE37448	Liver NK
GSM1585334	NK.CD49b+.Lv#2	GSE37448	Liver NK
GSM1585335	NK.CD49b+.Lv#3	GSE37448	Liver NK
GSM1585336	NK.LPL.NKp46+.SI#1	GSE37448	Small intestine NK
GSM1585337	NK.LPL.NKp46+.SI#3	GSE37448	Small intestine NK
GSM1945034	T4.SP.SPF#1	GSE75202	Spleen T4
GSM1945035	T4.SP.SPF#2	GSE75202	Spleen T4
GSM1945036	T8.SP.SPF#1	GSE75202	Spleen T8
GSM1945037	T8.SP.SPF#2	GSE75202	Spleen T8
GSM1945038	MF.PC.SPF#1	GSE75202	Spleen MF
GSM1945039	MF.PC.SPF#2	GSE75202	Spleen MF
GSM1945040	BB.SP.SPF#1	GSE75202	Spleen B
GSM1945041	BB.SP.SPF#2	GSE75202	Spleen B
GSM1945042	GN.SP.SPF#1	GSE75202	Spleen PMN
GSM1945043	GN.SP.SPF#2	GSE75202	Spleen PMN
GSM1945046	Treg.SP.SPF#1	GSE75202	Spleen Treg
GSM1945047	Treg.SP.SPF#2	GSE75202	Spleen Treg
GSM1945048	Tgd.SP.SPF#1	GSE75202	Spleen Tγδ
GSM1945049	Tgd.SP.SPF#2	GSE75202	Spleen Tγδ
GSM1945050	NK.SP.SPF#1	GSE75202	Spleen NK
GSM1945051	NK.SP.SPF#2	GSE75202	Spleen NK
GSM1945052	NKT.SP.SPF#1	GSE75202	Spleen NKT
GSM1945053	NKT.SP.SPF#2	GSE75202	Spleen NKT
GSM1945054	DC.SP.SPF#1	GSE75202	Spleen DC
GSM1945055	DC.SP.SPF#2	GSE75202	Spleen DC
GSM1945076	NK.SP.SPF#3	GSE75202	Spleen NK
GSM1945077	NKT.SP.SPF#3	GSE75202	Spleen NKT
GSM1945078	T4.SP.SPF#3	GSE75202	Spleen T4
GSM1945079	T8.SP.SPF#3	GSE75202	Spleen T8
GSM1945080	Treg.SP.SPF#3	GSE75202	Spleen Treg
GSM1945081	Tgd.SPF.SPF#3	GSE75202	Spleen Tγδ
GSM1945082	DC.SP.SPF#3	GSE75202	Spleen DC
GSM1945084	BB.SP.SPF#3	GSE75202	Spleen B
GSM1945085	MF.PC.SPF#3	GSE75202	Spleen MF
